# Honey Bees Reduce Pollen Viability While Foraging

**DOI:** 10.3390/insects17020199

**Published:** 2026-02-13

**Authors:** Alex C. Kurtt, Fernando de la Torre, Anna F. Edlund, Juan E. Zalapa, Shawn A. Steffan

**Affiliations:** 1 Department of Entomology, University of Wisconsin-Madison, Madison, WI 53706, USA; kurtt@wisc.edu; 2 Department of Plant and Agroecosystem Sciences, University of Wisconsin-Madison, Madison, WI 53706, USA; fdelatorre@wisc.edu (F.d.l.T.); juan.zalapa@usda.gov (J.E.Z.); 3 Department of Biology, Bethany College, Bethany, WV 26032, USA; aedlun@artic.edu; 4 Department of Liberal Arts, School of the Art Institute of Chicago, Chicago, IL 60603, USA; 5 US Department of Agriculture, Agricultural Research Service, Madison, WI 53706, USA

**Keywords:** pollen germination media, pollen basket, pollination, viability

## Abstract

Pollen acts as both a gametophyte for plant reproduction and a vital nutrient source for bees. Adult honey bees (*Apis mellifera*) mix pollen with nectar, enzymes, and microbes to create ‘bee bread’, diverting pollen from plant reproduction and re-appropriating it as larval food. However, the point at which corbicular pollen becomes nonviable is largely unknown. This question is important not only because it explicitly addresses pollen viability while bees pollinate, but also because it informs the food vs. fertilization tradeoff at the center of bee–angiosperm mutualisms. Here, we investigated changes in pollen viability during foraging bouts of honey bees. We observed pollen germination across two plant species: *Allium tuberosum* and *Solidago rigida*. Bee-collected pollen was contrasted against fresh pollen directly from floral anthers, de-ionized water-soaked pollen, and sucrose solution-washed pollen (a nectar substitute). The bee-collected pollen exhibited significant reductions in germination for both *A. tuberosum* and *S. rigida* pollen, compared to controls and the sucrose solution. Pollen viability, therefore, was greatly reduced while the bees in our study were foraging, suggesting that honey bees render pollen nonviable as they pollinate. These findings reveal why corbicular pollen contributes little to plant fertilization, highlighting the importance of non-corbicular ‘body pollen’.

## Simple Summary

Foraging honey bees consolidate pollen within ‘pollen baskets’ (corbiculae), dual structures on their hind legs that facilitate large pollen loads. The bees mix pollen with nectar and other compounds, creating a sticky mass that keeps the pollen affixed to their corbiculae. While foraging, pollen grains on the corbiculae may contact the floral stigma, but the viability of corbicular pollen is largely unknown. This question is important not only because honey bees are critical pollinators in agriculture globally, but also because the vast majority of collected pollen is consolidated on their corbiculae. Here, we examined corbicular pollen while honey bees were actively foraging. Such pollen exhibited significantly lower viability than pollen from the floral anther. Further, bee-collected pollen exhibited lower germination than pollen mixed with a nectar substitute, suggesting that compounds other than nectar sugars play a role in reducing viability. Thus, actively foraging honey bees rendered most of their pollen unavailable for plant fertilization, which explains why corbicular pollen contributes little to fertilization. This underscores the importance of non-corbicular pollen (‘body pollen’) in bee-mediated pollination.

## 1. Introduction

Angiosperms rely on pollinating insects as reproductive vectors to optimize the dispersal of pollen, thereby facilitating efficient cross-fertilization [1,2]. Given that relatively few pollen grains are needed for seed set, there is an immense surplus of pollen available for exploitation by pollinators and their microbial symbionts [2,3,4]. The harvest of pollen and nectar by bees is largely undertaken to provide food for their progeny and/or younger sisters [5]; thus, pollination may be viewed as a byproduct of larval brood care. Pollen can be conceptually categorized as either gametophytes destined for plant fertilization or as a bee dietary resource, with certain plant species producing pollen grains specific to each category (e.g., some plants produce infertile pollen grains that are known to be highly attractive to pollinators) [6].

The optimization of pollen collection and curation has given rise to a wide array of morphological and behavioral adaptations in bees. Setae, branched hair-like structures, cover the cuticle of virtually all bees, which increases the surface area for pollen contact and suspend pollen grains for easy removal [7]. The electrostatic potential between the pollen and setae is largely responsible for efficient pollen acquisition, while grooming behaviors and wetting via nectar regurgitation, allow corbiculate bees to concentrate pollen masses on their corbiculae [8]. This regurgitated nectar often includes enzymes, lactobacilli (lactic acid-forming bacteria), and cellulose-digesting yeasts that aid in pollen breakdown during fermentation, increasing its nutritional value to the hive [5,9,10] and its myriad microbial symbionts [11,12].

**Figure 1:**
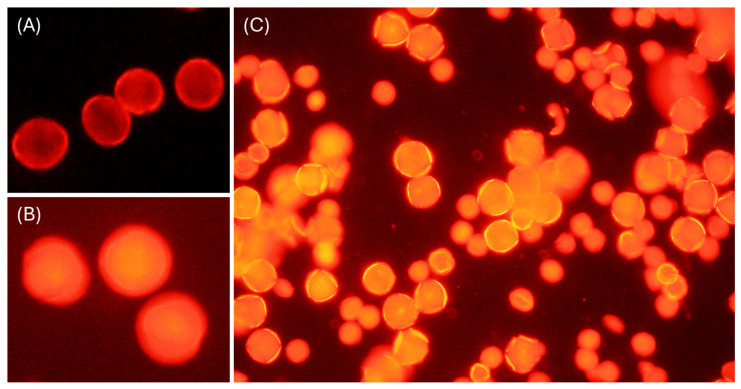
Fluorescent microscopy of pollen grains stained with 0.02 µg/mL propidium iodide suspended in 0.15 molar HEPES to examine viability. (**A**) Example of living pollen grains: exine is stained and brightly colored, while the cell contents are unstained and darker. (**B**) Example of nonviable pollen grains: both exine and cell contents are stained and highly fluorescent. (**C**) Sample of bee-collected pollen (from *Osmia lignaria* nest provisions) consisting almost exclusively of nonviable pollen grains.

The importance and ubiquity of bee-microbe symbioses have been frequently reported in honey bees, *Apis mellifera* L. [13], begging the question as to whether pollen grains destined for the hive would benefit from retaining reproductive function [4]. Based on repeated observations in previous work, pollen grains in larval pollen-provisions were entirely nonviable (Figure 1). Several studies have examined the viability of bee-collected pollen, with timescales ranging from less than a day to over a year [14,15]; however, to our knowledge, changes in pollen viability during a foraging bout have not yet been investigated. This is a critical knowledge gap, as it is during such foraging that pollen needs to be viable for fertilization. We address this question (pollen viability during foraging) using honey bees, *A. mellifera* L., a pollinator of profound importance globally.

Honey bees travel significant distances per foraging trip to collect adequate food for thousands of growing larvae in the hive [16]. Corbiculae are a specialized structure to optimize the transport of large pollen loads [8,17] and increase pollen collection per trip [18]. Having a means to carry more pollen per trip increases foraging efficiency, facilitating wider foraging ranges, yet the subsequent effects on plant fertilization efficiency may not be as clear. If pollen grains are rendered nonviable while honey bees are foraging (i.e., storing the pollen on their corbiculae), this could be problematic for plant reproduction and may undermine bee mutualisms with angiosperms.

Past research has demonstrated that pollen loaded onto corbiculae is less effective for pollination—specifically, corbicular pollen grains placed on plant stigmas were associated with significantly reduced seed set and fruit production [19]. Given that corbicular pollen is largely destined for the hive as food for larvae and microbes [5], changes in viability may not be inherently detrimental for the plant, as there is evidence that pollination is facilitated primarily through pollen grains on bees’ setae [19]. However, both corbicular and body-pollen contact plant stigmas. Considering its volumetrically (and numerically) high pollen abundance, corbicular pollen grains may still act as a reliable means of fertilization, unless grains are nonviable and thus unable to germinate. This formed the basis of our primary research questions: Are pollen grains capable of germinating after being consolidated and curated on corbiculae, and if so, to what degree? In essence, two of the major pollen destinations (hive vs. plant stigma) represent two distinct pathways (food vs. fertilization); however, it is unknown whether this delineation occurs before or after the bee returns to the nest.

Here, we have investigated a potential mechanism driving the divergence of the two primary pollen destinations/functions. To measure pollen viability, we quantified percent (%) germination from honey bee corbiculae vs. pollen directly from plant anthers where the bees had been foraging exclusively. Doing so should allow us to characterize pollen viability following collection and curation by foraging bees. Based on previous literature and empirical observation, we expect that bee-collected pollen will exhibit reductions in germination compared to untreated controls, pure water, and a nectar substitute. If pollen curated on corbiculae is rendered non-functional as gametophytes for plant fertilization, then this phenomenon may represent the cost–benefit relationships between honey bees and the angiosperms they visit.

## 2. Materials and Methods

### 2.1. Measurement of Pollen Viability

Quantifying pollen viability can be accomplished via several methods. The most prevalent methodologies are pollen staining and pollen germination media (PGM) culturing. Pollen staining (e.g., Propidium iodide, Alexander’s dye [20]) is applicable for a wide range of angiosperm taxa and is relatively straightforward; however, results are largely qualitative, and living versus inviable pollen grains are not always easily distinguishable. Additionally, the relative assessment of a pollen grain being intact does not guarantee its ability to germinate [21]. Utilizing PGM to induce germination is a more reliable metric for viability, as it directly measures germination by simulating the stigma of the flower [22]. This method is limited by the idiosyncrasies of stigma biochemistry and the difficulties associated with PGM optimization [23,24]. We assayed pollen germination via PGM in our study; however, neither staining nor PGM has been used to test viability in bee-collected pollen.

### 2.2. Site Description and Collections

Flowers and foraging *A. mellifera* workers were collected from a pesticide-free botanical garden (Allen Centennial Gardens) on the campus of the University of Wisconsin-Madison (Madison, WI, USA) in late September of 2024. Floral composition consisted of a blend of native prairie species and non-native ornamental taxa. *A. mellifera* workers with loaded pollen baskets were hand-netted from their flower and sealed in a 15 mL high-clarity polypropylene conical tube (Falcon tubes, Corning Science, Thermo Fisher, Madison, WI, USA). Specimens were placed in an ice-filled cooler to induce cold torpor. The bee’s most recently visited floret of the visited flower was removed at the stem and inserted into a 1.5 mL microcentrifuge tube (Avantor microcentrifuge tubes, VWR North America, Radnor, PA, USA) and labeled with the corresponding bee. Floral samples were stored within the cooler to maintain temperature consistency across treatments. If the majority of anthers in the collected floret had already dehisced (released their pollen), additional florets in anthesis from the visited plant were collected.

### 2.3. Development of Germination Media

Plant species were selected for PGM screening based on the visitation rates of *A. mellifera* foragers. Any dense stand of a flower species with high visitation (>10 bees on a given plant) represented a candidate for screening and subsequent treatments. To optimize in vitro germination, plant species were screened through multiple fully crossed two-way factorials of PGM recipes (Appendix A), based on published PGM recipes in previous literature [25,26,27]. The most successful factorial combination involved specific concentrations of boric acid (H_3_BO_3_, 0.005%, 0.01%, and 0.02% (*w*/*v*)) and sucrose (10%, 15%, and 20% (*w*/*v*)). Fresh pollen directly from anthers was tested for suitable levels of germination on PGM to establish the untreated control germination rate. Multiple plant species were screened to find the highest baseline germination rate using this PGM (see Appendix A for plant species tested and their respective percent germination rates). All culturing media, compounds, and plasticware were supplied by Thermo-Fisher (Madison, WI, USA).

The highest levels of germination were exhibited by *Allium tuberosum* and *Solidago rigida* with averages of 57.3% and 42.0%, respectively. Separate PGM recipes were used for each plant species to optimize germination in vitro. The PGM for *A. tuberosum* contained 15% sucrose (*w*/*v*), 0.005% H_3_BO_3_ (*w*/*v*), 0.066% CaCl_2_ · 2H_2_O (*w*/*v*), 0.005% KH_2_SO_4_ (*w*/*v*), and 0.5% Difco Agar (*w*/*v*). The *S. rigida* medium was identical but contained 20% sucrose (*w*/*v*). Both media were titrated with 0.25M NaOH, adjusting to neutral pH. While additional plants exhibited high percent germination, seasonal timing limited the number of usable species, as certain plants fell out of bloom before testing.

### 2.4. Treatments and Culturing

For each replicate of each plant species, pollen grains were collected directly from anthers and randomly aliquoted into three treatments: (1) a negative control (pollen alone), (2) a water-only treatment (pollen treated with de-ionized water), and (3) a sucrose treatment (pollen treated with an aqueous sucrose solution). The fourth treatment was pollen directly from the corbiculae of the honey bee that was foraging at the flowers corresponding to the anthers being tested for pollen viability. Each pollen sample was cultured independently on the plant-specific PGM. All pollen samples (N = 9 replicates/treatment) were cultured on PGM media within 100 × 15 mm Petri dishes. Within a given dish, the four treatments were randomly assigned to four separate quadrants: (1) control, (2) de-ionized water, (3) sucrose solution (10% sucrose in DI water), and (4) corbicular pollen from honey bees. The pollen samples were cultured at the center of each quadrant to maintain buffer space between quadrants, thereby ensuring that the treatments remained independent. Before culturing on the PGM, fresh pollen grains were deposited from the collected floret onto a glass slide for treatment application. The control pollen was collected on a camel hair brush and applied directly to the PGM. The DI water and sucrose solution (20 μL each) were mixed into separate sections of the fresh pollen, then brushed onto their respective quadrants. The pollen basket from the corresponding bee was removed and streaked into smaller densities before brushing onto the PGM. While pollen grains were not counted prior to incubation, pollen counts in any given sample were approximately 500–1500 grains, enough to facilitate random sub-sampling for estimates of percent germination per replicate.

**Figure 2:**
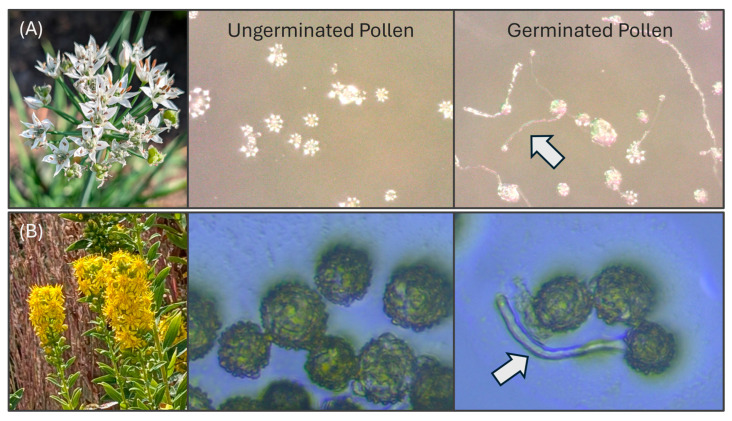
Comparisons of ungerminated and germinated pollen grains for the two species tested. The arrows indicate pollen tubes emerging from germinated pollen grains. (**A**) Garlic chives (*Allium tuberosum*). (**B**) Stiff goldenrod (*Solidago rigida*).

Replicates (individual Petri dishes with PGM) were stored at 22 °C for approximately 24 h. Post-incubation, the dishes were placed under an Olympus SZX16 light microscope to calculate germination. Within each replicate of each treatment, five sub-samples of ~50 pollen grains were randomly selected from different portions of the PGM substrate in a given quadrant of the petri dish. The numbers of germinated and ungerminated grains were counted within each sub-sample. The pollen was considered germinated when the pollen tube length exceeded the grain’s radius [26] (Figure 2). The germination rate (%) was derived by dividing the number of germinated pollen grains by the total number of pollen grains in a given sub-sample. The germination rate for a given replicate was the mean germination rate of the five sub-samples.

### 2.5. Statistical Analyses

All analyses were conducted in SigmaPlot (v.12). The effects of treatment on germination were assessed using a one-way ANOVA for each plant species. Where assumptions of either homoscedasticity (Levene’s test) or normality of data (Shapiro–Wilk test) were not met, a Kruskal–Wallis ANOVA test on ranks was performed. Pairwise comparisons (Tukey’s test for *A. tuberosum*; Holm–Sidak procedure for *S. rigida* germination) were conducted for all treatments.

## 3. Results

**Figure 3:**
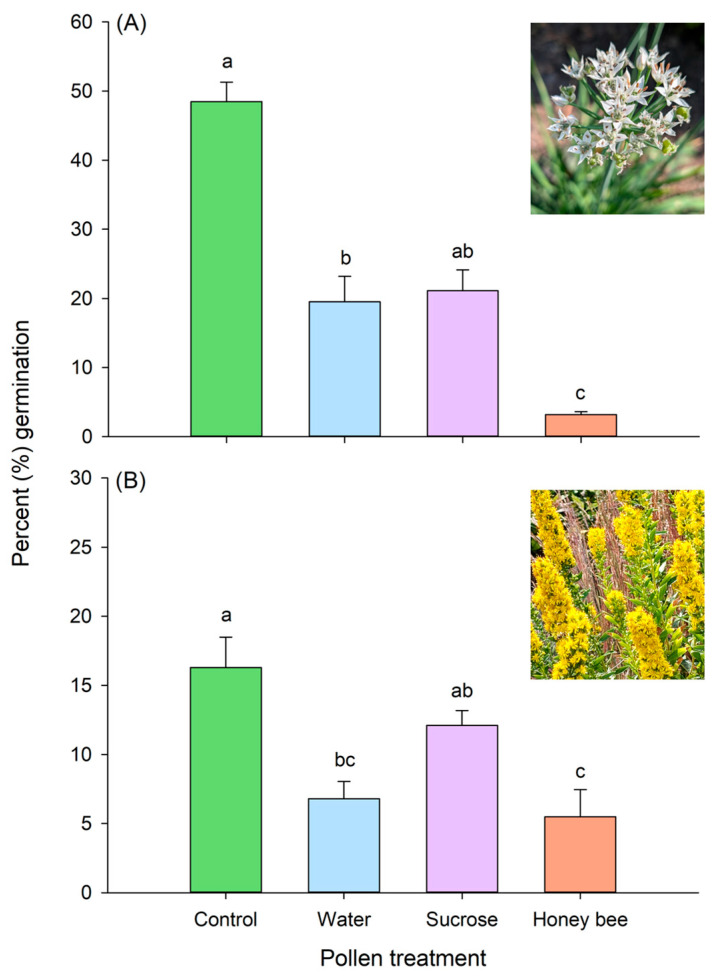
Percent germination of pollen grains within species-specific PGM for (**A**) *Allium tuberosum* (N = 9) and (**B**) *Solidago rigida* (N = 9) across treatments: control, de-ionized (DI) water, 10% sucrose solution (sucrose), and corbicular pollen from a honey bee. Different letters over each bar indicate significant differences (*p* < 0.05).

The treatments applied to pollen grains produced significantly different germination rates for both *A. tuberosum* (Kruskal–Wallis ANOVA on ranks: *H* = 29.13; df = 3; *p* < 0.001; Figure 3A) and *S. rigida* (one-way ANOVA: *F*_3, 24_ = 8.7; *p* < 0.001; Figure 3B).

Pairwise comparisons for *A. tuberosum* indicated that germination in the control treatment (median: 48.4%; IQR = 42.8–55.4%) was significantly greater (*q* = 7.62; *p* < 0.05) than that of the corbicular pollen treatment (median: 3.60%; IQR = 2.0–4.4%), as well as the DI water-treated pollen (median: 22.8%; IQR = 8.2–28.4%; *q* = 3.89; *p* < 0.05). The sucrose-treated pollen (i.e., nectar substitute) was not significantly different from the control (*p* > 0.05) for *A. tuberosum* but did germinate at significantly higher rates than corbicular pollen (*q* = 4.02; *p* < 0.05; IQR = 14.8–27.8%).

Similarly, pairwise comparisons for *S. rigida* indicated that the control germination (mean ± SE: 16.29 ± 2.20%) was significantly greater (*t* = 4.52; *p* < 0.001) than that of corbicular (5.49 ± 1.97%) and DI water-treated pollen (*t* = 3.97; *p* = 0.003; 6.80 ± 1.28%). The sucrose-treated pollen germination was not significantly different from the control (*p* > 0.5) but, as with *A. tuberosum*, had significantly higher germination rates than the corbicular pollen (*t* = 2.78; *p* = 0.04; 12.11 ± 1.07%). The sucrose-treated and the DI water-treated pollen were not significantly different for *S. rigida* or *A. tuberosum* (Figure 3).

## 4. Discussion

In the process of collecting and consolidating pollen grains on their corbiculae, honey bees significantly reduced the viability of *Allium tuberosum* and *Solidago rigida* pollen. Within hours of collection, the germination of bee-collected pollen was reduced 13- and 3-fold for *A. tuberosum* and *S. rigida,* respectively. Pure water also reduced pollen germination relative to controls, but to a lesser degree. Interestingly, the nectar simulation used to isolate potential osmotic gradient effects (sucrose solution) did not exhibit percent germination rates different from the controls for either plant species. Furthermore, when comparing the sucrose-treated pollen’s germination to that of honey bees, the sucrose germination was significantly higher, an effect consistent across both plant species. Bee manipulations of pollen consistently reduced pollen viability below that of nectar alone, suggesting that the sugar concentration of nectar does not fully account for the strongly reduced germination rates of pollen subjected to bee-mediated effects (e.g., enzymatic activity, microbial interactions, physical manipulation). Thus, in the act of pollinating, the honey bees in our study rendered the majority of their collected pollen nonviable.

Such reductions in the viability of corbicular pollen highlight the importance of body-pollen for pollination. In virtually all bee species, grooming behaviors facilitate the ‘harvest’ of body-pollen [28], and among the non-corbiculate bees (most bee species), this is accomplished when the foraging females have returned to their nests [8]. For corbiculate bee species, the harvesting of body-pollen is accomplished while the bee forages, concentrating pollen for efficient transport [8,17]; however, when corbiculate bees curate their pollen while foraging, this introduces the possibility that pollen viability may be compromised. Our findings suggest that honey bees do compromise their corbicular pollen; thus, pollination may derive primarily from the unharvested pollen grains on their setae (i.e., the result of imperfect grooming). Production of very small pollen grains likely increases the probability of pollen remaining unharvested on bee setae. Indeed, anisogamy allows gametophytes (pollen grains) to be produced in vast quantities at the expense of individual size [29,30], rendering each individual grain relatively expendable for the plant. Relying on corbiculate bees for gamete dispersal represents a highly successful tradeoff in which angiosperms benefit from bee-driven pollination, even though the vast majority of pollen is often sequestered by the bees [1,2].

By directly measuring germination following honey bee manipulation of pollen, our study provides evidence of a surprising phenomenon: honey bees render pollen grains nonviable as they pack it on their corbiculae. These findings provide a mechanistic explanation for the reductions in seed set reported in previous studies [19]. The implication of this phenomenon is that corbiculate bees, comprised largely of social species, may be less efficient (per capita) than non-corbiculate bee species. Conversely, most solitary species gather pollen on their scopa(e) and do not mix it with nectar [8]. The ‘free body-pollen’ on these non-corbiculate bees is more available for plant fertilization and has been shown to produce higher levels of seed set, despite being proportionately less abundant than corbicular pollen [19]. As a result, there may be elevated pollination costs for angiosperms being pollinated by corbiculate bees, such as honey bees. This conjecture is supported by our data, as well as others’, but we recommend follow-up studies comparing germination between body-pollen and corbicular pollen across a broader diversity of angiosperm and bee species. In essence, the remarkable capacity of setae to harvest pollen from an anther, coupled with the forager’s relative inability to extract all pollen from these setae, may facilitate adequate plant fertilization, even while the forager has loaded large amounts of pollen in the pollen basket. If corbicular pollen is effectively compromised in the process of curation, this likely represents an acceptable cost to the plant, assuming adequate fertilization is provided from body-pollen. Honey bees may be sequestering a greater proportion of plant pollen as food for their larvae, but this apparent asymmetry does not seem to have interrupted the stability, success, and/or persistence of their mutualism(s) with angiosperms [1,2].

## 5. Conclusions

Our results show that pollen grains within the honey bee pollen basket exhibit strongly reduced viability, suggesting that such pollen has been rendered nonviable by the foraging bee. Thus, while a honey bee is actively pollinating, the majority of its collected pollen is largely incapable of germinating, regardless of whether it contacts the plant stigma. It appears that honey bees not only commandeer the vast majority of collected pollen as food but also curate it immediately, in such a way that the pollen grains cannot effectively function as gametophytes. These findings, coupled with the attendant reductions in seed set [19], provide an explanation for why corbicular pollen contributes little to plant fertilization, which may represent a significant consequence for plant reproductive output. While the underlying mechanisms for this reduction in pollen viability remain largely unknown, our findings provide an ideal platform for further studies of the tradeoffs intrinsic to bee–microbe–angiosperm mutualisms.

## Data Availability

Data will be curated at the USDA Ag Data Commons (https://agdatacommons.nal.usda.gov/), accessed 10 February 2026.

## Appendix A

The tables below present fully crossed factorial combinations of ingredients (e.g., identity, rate, and composition) used in assays of pollen growth media (PGM). Each PGM type represented the means to establish a pollen viability baseline for a given plant species (using pollen grains directly from the floral anther). Percent (%) germination of pollen grains represented the metric used to assess the suitability of any given PGM.

**Table A1:** Factorial array of ingredients (boron × calcium concentrations) for evaluations of pollen germination media (recipes #1–9).

	0.5× Boron	1× Boron	1.5× Boron
0.5× Calcium	#1: 0.007% CaCl_2_, 0.012% Ca(NO_3_)_2_, 0.005% H_3_BO_3_, 0.025% MgSO_4_, 18% Sucrose, 0.5% Agar	#2: 0.007% CaCl_2_, 0.012% Ca(NO_3_)_2_, 0.01% H_3_BO_3_, 0.025% MgSO_4_, 18% Sucrose, 0.5% Agar	#3: 0.007% CaCl_2_, 0.012% Ca(NO_3_)_2_, 0.015% H_3_BO_3_, 0.025% MgSO_4_, 18% Sucrose, 0.5% Agar
1× Calcium	#4: 0.015% CaCl_2_, 0.024% Ca(NO_3_)_2_, 0.005% H_3_BO_3_, 0.025% MgSO_4_, 18% Sucrose, 0.5% Agar	#5: 0.015% CaCl_2_, 0.024% Ca(NO_3_)_2_, 0.01% H_3_BO_3_, 0.025% MgSO_4_, 18% Sucrose, 0.5% Agar	#6: 0.015% CaCl_2_, 0.024% Ca(NO_3_)_2_, 0.015% H_3_BO_3_, 0.025% MgSO_4_, 18% Sucrose, 0.5% Agar
1.5× Calcium	#7: 0.022% CaCl_2_, 0.035% Ca(NO_3_)_2_, 0.005% H_3_BO_3_, 0.025% MgSO_4_, 18% Sucrose, 0.5% Agar	#8: 0.022% CaCl_2_, 0.035% Ca(NO_3_)_2_, 0.01% H_3_BO_3_, 0.025% MgSO_4_, 18% Sucrose, 0.5% Agar	#9: 0.022% CaCl_2_, 0.035% Ca(NO_3_)_2_, 0.015% H_3_BO_3_, 0.025% MgSO_4_, 18% Sucrose, 0.5% Agar

**Table A2:** Pollen germination percentage (%) for plant species tested across PGM recipes #1–9 (outlined in Table A1).

PGM Recipe #	*Anethum* *graveolens*	*Lavendula* *augustifolia*	*Daucus carota*	*Gentiana alba*	*Solidago* *rigida*	*Phlox* spp.
1	1.9%	0%	0%	0%	1.4%	0%
2	0%	13%	2.3%	0%	0%	0%
3	0%	30%	1.6%	0%	0%	4%
4	0%	10%	0%	1.8%	1.6%	5.6%
5	3.4%	12.2%	1.2%	0%	0%	1.6%
6	0%	8.7%	1.6%	0%	4.1%	0%
7	0%	5.3%	0%	0%	0%	1.8%
8	0%	19%	0%	0%	0%	6.2%
9	2.1%	14.3%	0%	0%	0%	3.8%

**Table A3:** Factorial array of ingredients (sucrose × boron concentrations) for evaluations of PGM recipes #10–18.

	0.5× H_3_BO_3_	1× H_3_BO_3_	2× H_3_BO_3_
1× Sucrose	#10: 10% Sucrose, 0.005% H_3_BO_3_, 0.066% CaCl_2_ * H_2_O, 0.005% KH_2_SO_4_, 0.5% Agar	#11: 10% Sucrose, 0.01% H_3_BO_3_, 0.066% CaCl_2_ * H_2_O, 0.005% KH_2_SO_4_, 0.5% Agar	#12: 10% Sucrose, 0.02% H_3_BO_3_, 0.066% CaCl_2_ * H_2_O, 0.005% KH_2_SO_4_, 0.5% Agar
1.5× Sucrose	#13: 15% Sucrose, 0.005% H_3_BO_3_, 0.066% CaCl_2_ * H_2_O, 0.005% KH_2_SO_4_, 0.5% Agar	#14: 15% Sucrose, 0.01% H_3_BO_3_, 0.066% CaCl_2_ * H_2_O, 0.005% KH_2_SO_4_, 0.5% Agar	#15: 15% Sucrose, 0.02% H_3_BO_3_, 0.066% CaCl_2_ * H_2_O, 0.005% KH_2_SO_4_, 0.5% Agar
2× Sucrose	#16: 20% Sucrose, 0.005% H_3_BO_3_, 0.066% CaCl_2_ * H_2_O, 0.005% KH_2_SO_4_, 0.5% Agar	#17: 20% Sucrose, 0.01% H_3_BO_3_, 0.066% CaCl_2_ * H_2_O, 0.005% KH_2_SO_4_, 0.5% Agar	#18: 20% Sucrose, 0.02% H_3_BO_3_, 0.066% CaCl_2_ * H_2_O, 0.005% KH_2_SO_4_, 0.5% Agar

**Table A4:** Pollen germination % for plant species tested across PGM recipes #10–18 (outlined in Table A3).

PGM Recipe #	*Allium tuberosum*	*Solidago rigida*	*Hylotelephium telephium*	*Helianthus strumosus*
10	50%	14.7%	51%	6.7%
11	52%	12.7%	52.6%	7.3%
12	16.67%	22.7%	28%	9.3%
13	57.3%	20.7%	53.3%	17.3%
14	36%	19.3%	44.7%	10.7%
15	40.7%	18%	52.7%	20.7%
16	38%	42%	17.3%	14%
17	56.7%	27.3%	31.3%	20%
18	33.3%	30%	0%	33.3%
